# SUPPORTING CHINESE FAMILY CAREGIVERS OF RELATIVES WITH DEMENTIA——A PILOT INTERVENTION STUDY IN SHANGHAI

**DOI:** 10.1093/geroni/igad104.3610

**Published:** 2023-12-21

**Authors:** Ethan Liu, Xinyu Zhang, Jiahui Huang, Ansar Ayrat, Qiuling An, Fei Sun, Xuehan Zhang

**Affiliations:** Phillips Exeter Academy, Exeter, New Hampshire, United States; East China Normal University, Shanghai, Shanghai, China (People’s Republic); East China Normal University, Shanghai, Shanghai, China (People’s Republic); East China Normal University, Shanghai, Shanghai, China (People’s Republic); East China Normal University, Shanghai, Shanghai, China (People’s Republic); Michigan State University, East Lansing, Michigan, United States; Michigan State University, East Lansing, Michigan, United States

## Abstract

**Background:**

Effective interventions are urgently needed to assist dementia family caregivers in China, where limited formal support and services are available. This pilot study aims to test a psychoeducational program that consists of information, skills training, and the use of binaural sound through the Processing Inner Strength toward Actualization Therapy (PISTA) device to alleviate caregiver burden and improve mental health outcomes.

**Methods:**

Single subject design approach was used. Eight dementia family caregivers in Shanghai, China, were recruited and received a two-stage intervention over 12 weeks: Stage 1 (S1, Six 90-minute weekly sessions: Psychosocial education programs +PISTA) and Stage 2 (S2, Six 90-minute weekly sessions: Psychosocial education only). Caregiver outcomes, including caregiving burden, anxiety, depression, and positive aspects of caregiving (PAC), were assessed through established scales. Paired t-tests and visual analyses were used to compare outcome levels and trends between stages.

**Results:**

Seven participants completed the entire study, and 1 participant dropped out during S2. Participants showed a decrease in caregiver burden, anxiety, and depression and an increase in PAC during S1. A similar trend was observed during S2 on burden, anxiety, and PAC outcomes, except for depression. Furthermore, the decrease in burden and anxiety scores and the increase in PAC scores were less salient during S2 than during S1, suggesting more effects were observed in S1 with PISTA.

**Conclusion:**

Evidence is promising that psychoeducational programs combined with PISTA can lead to better psychological outcomes for dementia family caregivers. Findings need to be affirmed in future investigations using an experimental design.

